# Foreword: An educational reference for pulsed field ablation

**DOI:** 10.1093/europace/euag245

**Published:** 2026-10-21

**Authors:** Samuel J Asirvatham, Tomás García-Sánchez, Jacob Koruth, Kars Neven, Andrea Sarkozy

**Affiliations:** Department of Cardiovascular Diseases, Mayo Clinic, 200 1st Street SW, Rochester, MN, USA; Medical Education EMEA, Boston Scientific Corporation, Madrid, Spain; Helmsley Electrophysiology Center, Mount Sinai Fuster Heart Hospital, Icahn School of Medicine at Mount Sinai, New York, NY, USA; Department of Electrophysiology, Alfried Krupp Hospital, Essen, Germany; Department of Medicine, Witten/Herdecke University, Witten, Germany; Heart Rhythm Management Centre, Postgraduate Program in Cardiac Electrophysiology and Pacing, Universitair Ziekenhuis Brussel—Vrije Universiteit Brussel, European Reference Networks Guard-Heart, Brussels, Belgium

Within only a few years, pulsed field ablation (PFA) has evolved from an emerging concept to a therapeutic modality adopted worldwide and increasingly considered to be a central component of modern catheter ablation. Its rapid adoption has generated tremendous interest among clinicians, researchers, engineers, and industry who share a common goal: improving the safety, efficacy, and reproducibility of catheter ablation procedures.

As with many transformative technologies, however, clinical adoption has progressed faster than the consolidation of knowledge. While the number of scientific publications on PFA has dramatically increased, clinicians, researchers, engineers, and trainees seeking to understand PFA often face a fragmented body of literature scattered across multiple disciplines, and it is not possible to find a single educational resource to learn about all aspects of PFA technology.

The idea behind this Europace Supplement was born from the recognition of this challenge. Our objective was simple but ambitious: to bring together, in one place, the current scientific understanding of PFA and create the first complete comprehensive educational reference for the electrophysiology community.

The result is what we believe to be the first educational resource specifically designed to provide a complete overview of PFA mechanisms, technology, tissue interactions, procedural and clinical considerations, and emerging developments.

Our objective was to create a comprehensive educational resource that becomes a resource to which readers return repeatedly throughout their professional journey. For experienced operators, a deeper understanding of these mechanisms can support more informed clinical decision-making and facilitate critical evaluation of emerging technologies. For physicians beginning their journey with PFA, the supplement provides a structured introduction to the scientific principles behind the therapy. For fellows, researchers, and allied professionals, it offers a framework that connects engineering principles with clinical practice and creates a common language for the entire scientific and clinical communities. We hope that readers from all levels of experience will find relevant and practical knowledge within these pages.

The educational value of this supplement deserves particular emphasis. Understanding why PFA behaves differently from thermal ablation technologies requires breaking barriers, clarifying misconceptions and accessing new knowledge that extends beyond traditional electrophysiology training. The contents of the supplement follow a logical progression. The opening articles establish the foundations of PFA, ranging from cell membrane electroporation, system design and technological concepts to differential tissue effects, lesion formation, or interactions with surrounding tissues. Subsequent contributions examine in detail the mechanisms and procedural aspects that underpin both efficacy and safety. The final articles focus on clinical aspects aimed at optimizing the therapy, ranging from sedation and anaesthesia strategies and approaches to the treatment of atrial fibrillation, to current perspectives of PFA and Left atrial appendage closure, as well as the future evolution of PFA technologies and applications.

Readers will notice that the supplement does not attempt to provide definitive answers to every question. On the contrary, many chapters highlight areas where knowledge remains incomplete. The field continues to evolve rapidly, and several scientific, technical, and clinical questions remain open. We believe that acknowledging uncertainty is an important component of scientific education. By identifying current knowledge gaps, we hope this publication will not only educate but also stimulate future investigation.

The multidisciplinary nature of the contributing authors also deserves special attention. The development of PFA has been driven not only by electrophysiologists, but also by experts in electroporation science, biomedical engineering, and translational research. Bringing these perspectives together was essential to creating a complete and balanced educational resource. Many of the most important questions surrounding PFA can only be answered through collaboration between disciplines, and this supplement reflects that philosophy.

We would like to express our sincere gratitude to all authors for their dedication, expertise, and commitment to this project. The development of a comprehensive educational resource of this scope requires substantial effort and collaboration. We are deeply appreciative of their contributions and their willingness to share their knowledge and unvaluable experience with the wider scientific community.

Looking forward, PFA is expected to continue its evolution through improvements in waveform design, catheter technologies, dosing strategies, treatment personalization, and novel clinical applications. At the same time, the need for high-quality education will only increase. Our aspiration is that this supplement becomes a reference resource that supports learning and discussion and serves as a catalyst for continued innovation, research and optimized clinical practice to ultimately improve patient care.

Finally, the Guest Editors thank Boston Scientific for supporting this initiative and their vision of creating an accessible, high-quality educational reference on PFA. The scientific content, interpretations, and conclusions presented in the supplement remain the responsibility of the authors and Guest Editors.

